# Identification and characterization of signature genes related to fetoplacental vascular endothelial cell programming in gestational diabetes mellitus using bioinformatics analysis

**DOI:** 10.3389/fgene.2025.1600756

**Published:** 2025-07-14

**Authors:** Chunhong Liu, Caicheng Wei, Yulan Lu, Fu Chai, Chunfang Wang, Yonglong Zeng, Huatuo Huang

**Affiliations:** ^1^ Center for Medical Laboratory Science, Affiliated Hospital of Youjiang Medical University for Nationalities, Baise, China; ^2^ Key Laboratory of Research on Clinical Molecular Diagnosis for High Incidence Diseases in Western Guangxi of Guangxi Higher Education Institutions, Baise, China; ^3^ Key Laboratory of Research and Development on Clinical Molecular Diagnosis for High-Incidence Diseases of Baise, Baise, China; ^4^ Department of Medical Reproduction Center, Affiliated Hospital of Youjiang Medical University for Nationalities, Baise, China

**Keywords:** gestational diabetes mellitus, fetoplacental vascular endothelial cells, fetus, weighted gene co-expression network analysis, bioinformatics analysis

## Abstract

Gestational diabetes mellitus (GDM) is a common pregnancy-related disorder with potential impacts on the fetoplacental unit. To uncover the underlying molecular mechanisms, we conducted a comprehensive bioinformatics analysis using a dataset from Gene Expression Omnibus, which included 37 primary human fetoplacental vascular endothelial cells (FPVEs) from healthy and GDM-complicated pregnancies. We identified 613 differentially expressed genes (DEGs) through the limma package, with 260 up-regulated and 353 down-regulated. Weighted gene co-expression network analysis was then performed, clustering genes into 11 modules. The MEdarkgreen module, containing 1,391 co-expression genes, showed the highest correlation with FPVE programming. After intersecting with DEGs, 192 co-expression hub genes were obtained. Gene Ontology enrichment analysis of these hub genes revealed enrichment in biological processes such as ribonucleoprotein complex biogenesis and ncRNA processing. Kyoto Encyclopedia of Genes and Genomes (KEGG) pathway analysis showed significant enrichment in pathways related to ribosome function, neurodegenerative diseases, and oxidative phosphorylation. Protein-protein interaction network analysis led to the identification of five signature genes (RPS13, MRPS5, MRPL22, MRPL21, and NDUFS3). These genes exhibited significantly lower expression in FPVEs from GDM pregnancies and demonstrated excellent diagnostic performance, with high area under the curve values in receiver operating characteristic analysis. Further KEGG signaling pathway analysis elucidated the multiple signaling pathways in which these signature genes are involved under GDM conditions. We also constructed LncRNA-miRNA-target genes interaction networks for the signature genes. The networks showed that the expression of these genes is regulated by multiple miRNAs and LncRNAs, highlighting the complex post-transcriptional regulatory mechanisms at play. Overall, our study provides novel insights into the molecular basis of FPVE programming in GDM and potential biomarkers for its diagnosis and understanding.

## Introduction

Gestational diabetes mellitus (GDM) is a form of diabetes that first appears during pregnancy. It typically develops in the second half of pregnancy, most commonly at 24–28 weeks (Szmuilowicz et al., 2019). The global prevalence of GDM varies significantly, ranging from 1% to over 30% (Szmuilowicz et al., 2019). The Middle East and North Africa (median 15.2%) and Southeast Asia (15.0%) have the highest rates, while Europe (6.1%) and North America and the Caribbean (7.0%) report relatively lower figures. In China, the prevalence of GDM shows a significant upward trend with marked regional disparities (data from WHO, from 2005 to 2018). Using the 2011 criteria of the International Association of Diabetes and Pregnancy Study Groups, the pooled prevalence of GDM in mainland China is 14.8%, with higher rates in economically developed eastern and southern regions (e.g., Qingdao in Shandong Province 21.8%, Guangdong Province 22.94%) and lower rates in western and northwestern areas (e.g., Xinjiang 5.12%) (Gao et al., 2019). Its core mechanism involves the interplay between insulin resistance and pancreatic β-cell dysfunction: Hormones secreted by the placenta during pregnancy (such as human placental lactogen and estrogen) induce insulin resistance in peripheral tissues (skeletal muscle and adipose tissue), while placenta-derived inflammatory cytokines (e.g., TNF-α) disrupt insulin signaling (Szmuilowicz et al., 2019). In women with pre-existing genetic susceptibility (e.g., TCF7L2, MTNR1B gene variants) or metabolic abnormalities (obesity, prediabetes), pancreatic β-cells fail to compensate for increased insulin resistance through enhanced secretion, leading to elevated fasting and postprandial blood glucose levels (Zhang et al., 2013; Kwak et al., 2012; Plows et al., 2018). Additionally, placental glucose transport dynamics, fetal hyperinsulinemia feedback, and environmental factors (high-fat diet, physical inactivity, environmental pollutants) further exacerbate the condition, culminating in clinical symptoms during mid-to-late pregnancy (Szmuilowicz et al., 2019). GDM is not only a concern during pregnancy but also has long-term implications for both the mother and the offspring (Farahvar et al., 2019). Maternal risks associated with GDM include an increased likelihood of developing type 2 diabetes later in life, as well as a higher risk of pre-eclampsia and other pregnancy-related complications (Moon and Jang, 2022). For the fetus, GDM can lead to macrosomia, which may result in difficulties during delivery, neonatal hypoglycemia, and an elevated risk of obesity and diabetes in childhood and adulthood (Farahvar et al., 2019; Moon and Jang, 2022). Currently, complications in offspring from mothers with GDM have garnered significant global attention, as the prenatal and postnatal periods represent critical windows that profoundly influence an individual’s long-term development and health outcomes. Nevertheless, despite substantial research efforts, the mechanistic pathways through which maternal GDM contributes to these offspring complications remain incompletely elucidated.

The fetoplacental unit is a complex and dynamic system that plays a crucial role in maintaining a healthy pregnancy. FPVEs are at the forefront of the maternal-fetal interface, responsible for regulating the exchange of nutrients, oxygen, and waste products (Covarrubias et al., 2023). These cells also play a vital role in the development of the placenta and the overall growth and development of the fetus. In the context of GDM, the normal function of FPVEs may be disrupted, leading to adverse pregnancy outcomes (Cvitic et al., 2018). Previous studies have revealed the association between FPVE dysfunction in GDM and pregnancy complications from multiple dimensions. In the oxidative stress pathway, the downregulation of the Nrf2 antioxidant system and miR-142-5p-mediated Nrf2 dysregulation can lead to FPVE damage, thereby inducing fetal vascular dysfunction (Yin et al., 2022; Milan et al., 2024). Insulin signaling defects exacerbate endothelial metabolic disorders by affecting adenosine transport and the IR-A pathway (Guzmán-Gutiérrez et al., 2014; Sobrevia et al., 2016). Placenta-derived exosomes carrying miRNAs (e.g., miR-140-3p, miR-130b-3p) interfere with angiogenesis by targeting molecules such as Chemerin and ICAM-1, while abnormal transmission of exosomes in the maternal-fetal circulation is associated with preeclampsia-like symptoms (Zhang et al., 2022; Gao et al., 2022; Salomon et al., 2016). At the epigenetic level, AngiomiR expression memory effects, fetal sex-specific miRNA profile differences, and dysregulation of lncRNA are involved in FPVE functional remodeling (Strutz et al., 2021; Strutz et al., 2018; Wang et al., 2019). Regardless of these advancements, the molecular mechanisms underlying the impact of GDM on FPVEs and the consequences of FPVE dysfunction on fetal complications remain poorly understood.

High-throughput gene expression profiling technologies, such as microarray analysis, have provided a powerful means to study the molecular changes associated with various diseases, including GDM (Uesaka et al., 2022). By analyzing gene expression data from FPVEs in normal and GDM-affected pregnancies, it is possible to identify DEGs that may be involved in the pathophysiology of GDM. Weighted gene co-expression network analysis (WGCNA) can help in uncovering gene modules that are co-expressed and associated with specific clinical traits, such as GDM-related changes in FPVE function (Zhao and Li, 2019; Langfelder and Horvath, 2008).

Once candidate genes are identified, functional annotation through Gene Ontology (GO) and KEGG pathway enrichment analyses can provide insights into the biological functions and pathways in which these genes participate (Yu et al., 2012). Further protein-protein interaction (PPI) network analysis based on the candidate genes is not only essential for understanding how the proteins encoded by these genes interact with each other, but also helps to identify critical genes of the network (Szklarczyk et al., 2019). Additionally, exploring the regulatory networks of these genes, such as the LncRNA-miRNA-target gene interactions, can reveal the complex post-transcriptional regulatory mechanisms at play in FPVEs under GDM conditions.

Therefore, we aimed to comprehensively analyze a publicly available gene expression dataset from FPVEs in normal and GDM-complicated pregnancies. By applying a series of bioinformatics analyses, we sought to identify signature genes related to FPVE programming in GDM, as well as to elucidate their functions, signaling pathways, and regulatory networks. This research may contribute to a better understanding of the molecular mechanisms of GDM and potentially identify novel biomarkers for its diagnosis and therapeutic targets for its management.

## Materials and methods

### Data source

**FIGURE 1:**
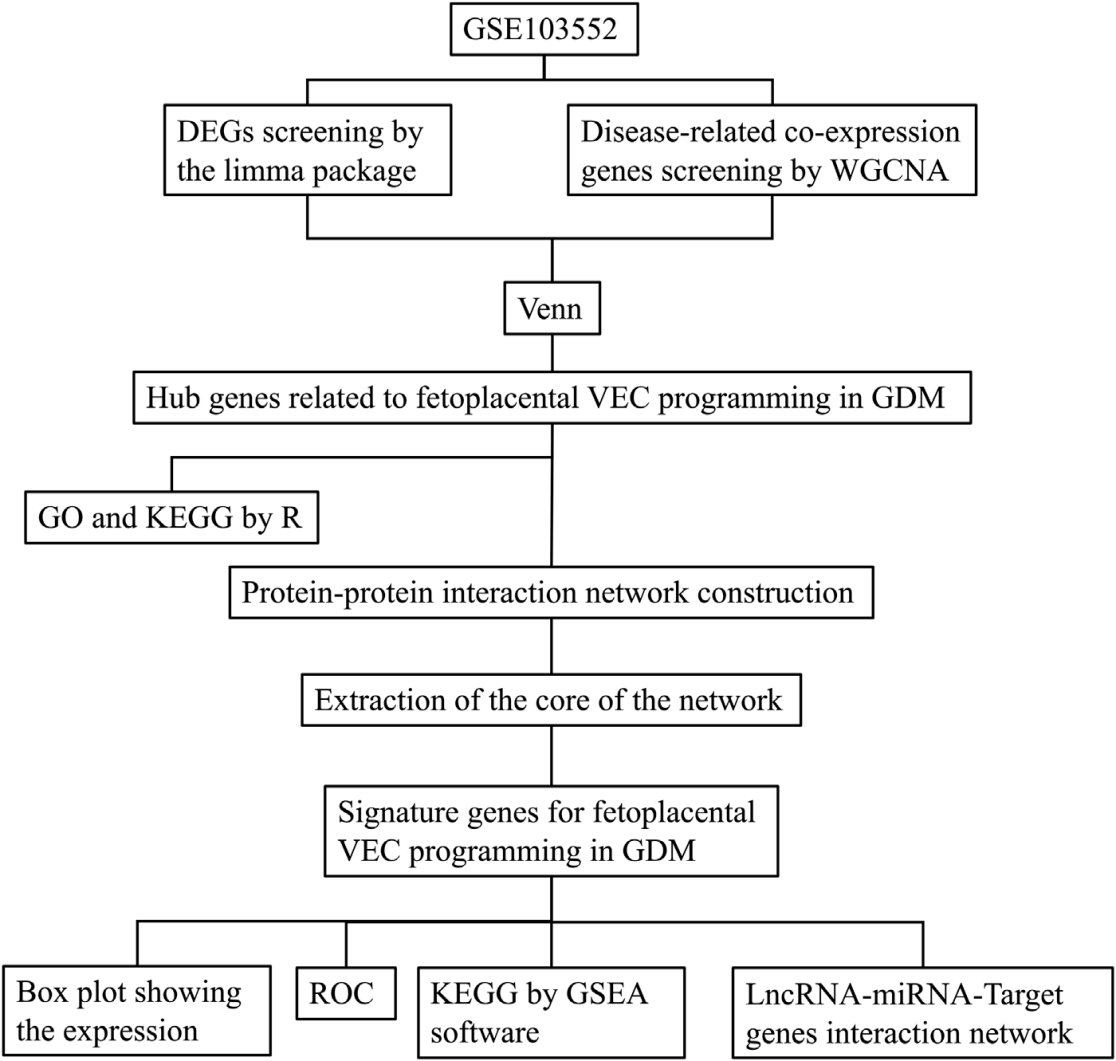
The experimental protocol of the study. DEGs, Differentially expressed genes; WGCNA, Weighted correlation network analysis; VEC, Vascular endothelial cell; GDM, Gestational diabetes mellitus; GO, gene ontology; KEGG, Kyoto encyclopedia of genes and genomes; ROC, receiver operating characteristic; GSEA, Gene set enrichment analysis; LncRNA, Long noncoding RNA; miRNA, MicroRNA.

The dataset [GSE103552 (Platform: GPL6244; Affymetrix Human Gene 1.0 ST Array)] used in this study was obtained from Gene Expression Omnibus (GEO) (https://www.ncbi.nlm.nih.gov/geo/). In this dataset, a total of 37 primary human FPVEs from arteries and veins were isolated after a healthy pregnancy (8 arteries, 8 veins) and after a pregnancy complicated by GDM (11 arteries, 10 veins). Our study focused on the global impact of GDM on the functional properties of fetoplacental vascular endothelial cells rather than comparing heterogeneity between arterial and venous endothelial cells. Therefore, we combined data from both cell types to comprehensively characterize the overall effects of GDM on the fetoplacental vascular endothelial system while avoiding reduced statistical power due to fragmented sample sizes. The study flow chart is shown in Figure 1.

### Principal component analysis and identification of differentially expressed genes

Principal component analysis (PCA) was performed using the prcomp function in R to explore potential structures and grouping characteristics among samples. We first read the gene expression matrix and grouping information, followed by log2 (x + 1) transformation, centering, and scaling of the expression matrix. The first two principal components (PC1 and PC2) were extracted, and their variance explained ratios were calculated. A two-dimensional scatter plot was generated using the ggplot2 package, with PC1 and PC2 as axes, where samples were colored and shaped by group, and 95% confidence ellipses were added to visualize group distributions. Additionally, the top 10 genes contributing most to PC1 and PC2 were identified to highlight key variables driving sample separation. Results were output as a PCA scatter plot. After that, identification of DEGs was performed using the limma package. In detail, gene expression data were preprocessed, including duplicate removal, normalization, and log2 transformation as needed. Differential expression analysis between healthy controls and GDM groups was performed using the limma package, with thresholds of |log2 fold-change| > 0.3 and adjusted p-value < 0.05 to identify significant genes. Results were visualized with a heatmap of the top 50 upregulated and downregulated genes and a volcano plot highlighting significant changes. Key results and normalized data were exported for further analysis.

### Identification of disease-related co-expression genes by WGCNA

We conducted WGCNA to identify gene modules associated with clinical traits using normalized expression data. After preprocessing, including the removal of genes with standard deviation ≤ 0.1 and outlier samples based on a clustering height cutoff of 20,000, we constructed a scale-free network with an optimal soft-thresholding power determined by the scale-free topology model fit (R^2^ ≥ 0.8). Gene modules were identified using a dynamic tree-cutting algorithm with a minimum module size of 60 and were merged based on eigengene correlation at a dissimilarity threshold of 0.25. Correlation analysis was performed to assess module-trait relationships, visualized using heatmaps. Gene significance (GS) and module membership (MM) were calculated, and hub genes were identified using thresholds of GS > 0.5 and MM > 0.8. All significant findings, including module-specific and hub genes, were documented, and key plots were saved for further analysis. Differentially expressed hub genes were obtained by intersecting genes from DEGs and WGCNA using the Venn diagram.

### GO analysis of the differentially expressed hub genes

We performed GO enrichment analysis to explore the functional annotations of intersected genes. First, we processed the input gene list, filtering out genes without Entrez IDs. GO enrichment analysis was conducted using the enrichGO function from the clusterProfiler package with parameters set to a p-value cutoff of 1 and a q-value cutoff of 1. Only significantly enriched terms meeting the criteria of p-value < 0.05 and q-value < 1 were retained. Enrichment results were visualized through various plots, including bar plots and bubble plots, highlighting the top 10 categories for each ontology (Biological Process, Cellular Component, and Molecular Function). Additionally, a GO circle plot was generated to depict the enrichment results using a customized color scheme based on ontology categories and p-value significance. All analyses were implemented in R with a reproducible workflow, and the output, including plots and enriched terms, was saved for further interpretation.

### KEGG analysis of the differentially expressed hub genes

We conducted the KEGG pathway enrichment analysis to investigate the biological pathways associated with intersected genes. The input gene list was processed by mapping gene symbols to Entrez IDs, and genes without corresponding Entrez IDs were excluded. KEGG enrichment analysis was performed using the enrichKEGG function from the clusterProfiler package with parameters set to a p-value cutoff of 1 and a q-value cutoff of 1. Pathways meeting the significance thresholds of p-value < 0.05 and q-value < 1 were retained for downstream analysis. The results were visualized using bar plots and bubble plots, displaying the top 30 enriched pathways or fewer if the total number of significant pathways was less than 30. The pathway descriptions were formatted for clarity, and all enriched pathways and visualizations were saved for further interpretation. Analyses were conducted in R with reproducible scripts.

### Further screening of the signature genes by protein-protein interaction network

Hub genes identified from the intersection of DEGs and WGCNA were analyzed in the database, namely, STRING (https://cn.string-db.org/). Briefly, after inputting the gene list and organism selection, the initial protein network was generated. For the setting of the network, the network type was set as full STRING network, meaning of network edges as evidence, active interaction sources as select all, minimum required interaction score as 0.4, and network display options as hide disconnected nodes in the network. After that, the protein interaction network was exported as a high-resolution bitmap. Moreover, the network was exported as a short tabular text output for further abstraction of the core of the network. The core of the network was abstracted in the Cytoscape software (Version: 3.10.2) using the tool CytoHubba based on the calculated Node’s score. The node’s scores were exported. Finally, the top 5 nodes ranked by degree were abstracted as the core of the network and saved as an image.

### Box plot and receiver operating characteristic (ROC) curve showing the expression and diagnostic performance of the signature genes

Differential expression and ROC analysis were conducted. Expression data were normalized using the normalizeBetweenArrays function, with log2 transformation applied to datasets with large numeric ranges (e.g., 99th percentile > 100 or range > 50 with a 25th percentile > 0). Expression data for control and treatment groups were integrated, with samples labeled accordingly. Target genes were extracted based on an intersection gene list. Differential expression analysis was performed, and box plots were generated using ggpubr to compare gene expression between groups, with statistical significance assessed. The top significant genes were visualized. ROC curves were plotted for each intersected gene using the pROC package. The area under the curve (AUC) and 95% confidence intervals (calculated by bootstrap) were displayed on the ROC plots, allowing the evaluation of gene classification performance. All analyses were conducted in R with reproducible scripts.

### The KEGG signaling pathway analysis for the signature genes

The KEGG signaling pathway analysis was performed using GSEA software (V4.3.2). Briefly, GCT and CLS files for each signature gene were prepared using a Perl script. The script calculates the median expression value of the gene and categorizes samples as having high (h) or low (l) expression based on this median. Two output files are generated: a GCT file containing expression data for all genes and a CLS file classifying samples into high and low-expression groups. These files are formatted to be compatible with the GSEA software for downstream analysis. Then, the GCT file was loaded into GSEA under the conditions including gene sets database as kegg_medicus, phenotype labels as h_versus_l, and collapse/Remap to gene symbol as No_collapse. Afterward, the top 10 high and low enrichment pathways with a NOM p-value < 0.05 were selected and grouped by an R script. The script reads all .tsv files from each signature gene and combines them into a single data frame, and assigns pathway names based on the names of the pathways. It generates two main visualizations: a line plot of running enrichment scores for each pathway and a heatmap of gene rankings across pathways. Custom color palettes and refined plot aesthetics are applied using the ggplot2 package. The two plots are aligned and combined into a single layout using gridExtra, ensuring consistent formatting.

### LncRNA-miRNA-target genes interaction network of the signature genes

To construct the interaction network based on the identified signature genes, we first predicted miRNAs for the signature genes by screening three miRNA databases, namely, miRanda (http://www.microrna.org/microrna/home.do), miRDB (https://mirdb.org/), and TargetScan (https://www.targetscan.org/vert_80/). Only those miRNAs that appeared in at least two databases simultaneously were included in the analysis. After that, targeted LncRNAs that have an interaction with the predicted miRNAs were identified on the spongeScan (http://conesalab.org/spongescan-a-web-for-detecting-microrna-binding-elements-in-lncrna-sequences/). The interaction network that shows the intricate interaction of the predicted miRNAs and LncRNAs for the signature genes was generated using Cytoscape (Version: 3.10.2).

## Results

### PCA analysis and DGEs identification of the dataset

**FIGURE 2:**
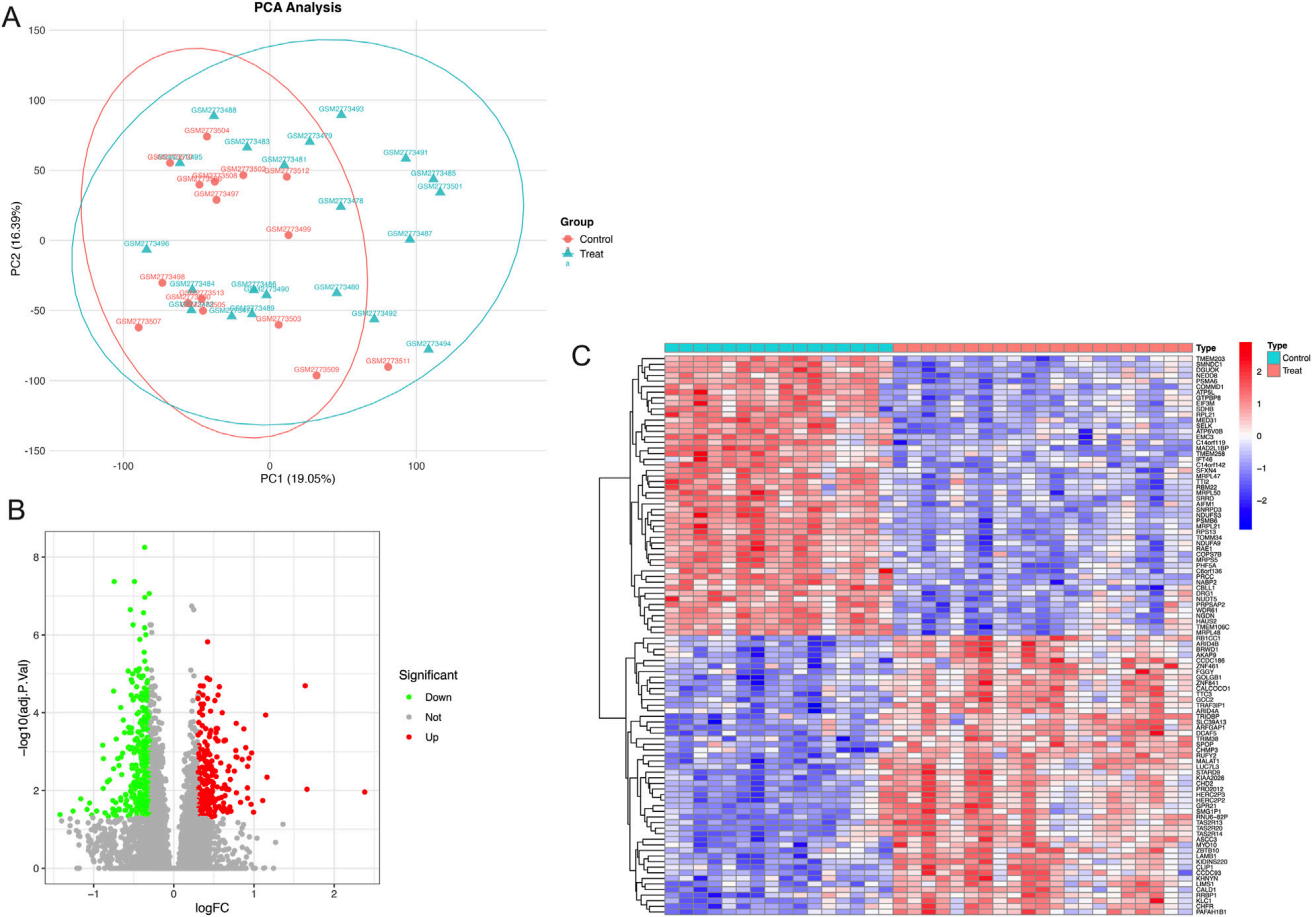
Principal component analysis and differentially expressed genes related to fetoplacental programming. **(A)** Principal component analysis of the dataset, **(B)** Differentially expressed genes displayed by a volcano diagram, and **(C)** 50 up and 50 downregulated differentially expressed genes displayed by a heatmap.

PCA analysis of the dataset was performed, and the results are shown in Figure 2A. In the PCA scatter plot, the horizontal axis PC1 has a variance explanation rate of 19.05%, and the vertical axis PC2 has a variance explanation rate of 16.39%. Different shapes and colors are used to distinguish the control group (triangles) and the GDM group (circles). There are also ellipses representing the 95% confidence interval. As can be seen from the graph, there is a separation trend between the two groups of samples in the PC1 and PC2 dimensions, indicating that the inter-group differences can be well distinguished. The dispersion degree of samples can be evaluated by the size of the ellipses. Outliers such as GSM2773499 can also be identified in Figure 2A. A total of 613 DEGs were found based on the cutoff criteria, with 260 of these genes being upregulated while 353 were downregulated. The volcano plot in Figure 2B shows these upregulated and downregulated DEGs. The heatmap in Figure 2C shows the details of the top 50 upregulated and top 50 downregulated DEGs. The details of all DEGs can be seen in the Supplementary Material.

### WGCNA for screening co-expression genes related to FPVE programming

**FIGURE 3:**
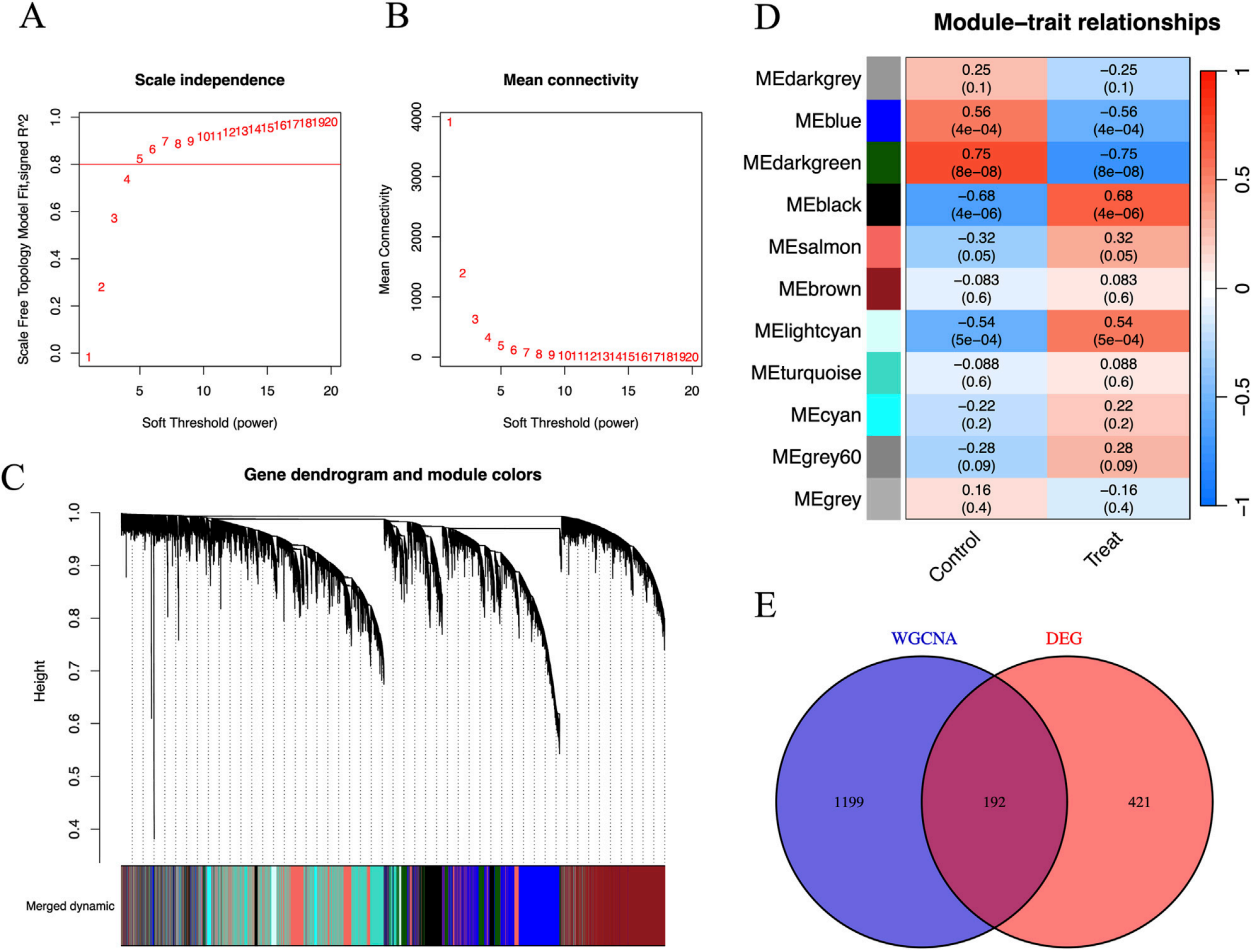
Identification of disease-related co-expression genes by WGCNA. **(A)** Scale-Free Topology Model Fit vs. Soft Threshold Plot to determine the optimal soft threshold power to make the constructed gene co-expression network have scale-free characteristics, **(B)** Mean Connectivity vs Soft Threshold Plot to understand the impact of different soft thresholds on the sparsity of the network, **(C)** Gene Dendrogram and Module Assignment Plot, **(D)** The Gene Modules and Traits for WGCNA, and **(E)** The interaction between DEGs and genes in the MEdarkgreen module by Venn diagram. DEGs, Differentially expressed genes; WGCNA, Weighted correlation network analysis.

Figures 3A,B show the relationship between the soft threshold (power value) and the scale-free topology model goodness-of-fit (signed R^2^), as well as mean connectivity. Figure 3A demonstrates that as the soft threshold increases, signed R^2^ first rises and then plateaus from 5 to 20, reflecting the degree to which the node degree distribution fits the power-law model. Figure 3B indicates that the mean connectivity decreases with increasing soft threshold. The significance of creating this plot lies in selecting a soft threshold that allows signed R^2^ to reach a plateau and meet the requirements, balancing network sparsity and connectivity to construct a gene co-expression network with scale-free properties, which lays the foundation for subsequent module identification and hub gene analysis. We can see that the optimal soft threshold was at 5. Figure 3C shows the gene dendrogram and modules labeled in different colors for WGCNA. Figure 3D shows the association analysis of 11 modules (named with ME + color, such as MEblue) with phenotypes (Control/Treat). By calculating the Pearson correlation coefficient (r) and significance (p-value) between the module eigengene (the first principal component of gene expression within the module) and phenotypes, the results are visualized with phenotypes on the horizontal axis and correlation coefficients on the vertical axis. The height of bars or position of points reflects the value of r, and the p-value is indicated in parentheses. Modules are distinguished by color or labels. Figure 3D identifies core modules significantly associated with phenotypes (e.g., MEdarkgreen with Treat group: r = 0.68, p = 4 × 10^−6^), providing directions for revealing the regulatory mechanisms between gene networks and phenotypes. We can see that genes from the dataset were clustered into 11 modules, and the MEdarkgreen module, which included 1,391 co-expression genes, exhibited the highest correlation and the most significant difference (cor = −0.75; p = 8e-08) among these modules (Figure 3D). After intersecting with genes from DEGs, 192 co-expression hub genes related to FPVE programming were identified (Figure 3E).

### GO and KEGG analyses of the co-expression hub genes

**FIGURE 4:**
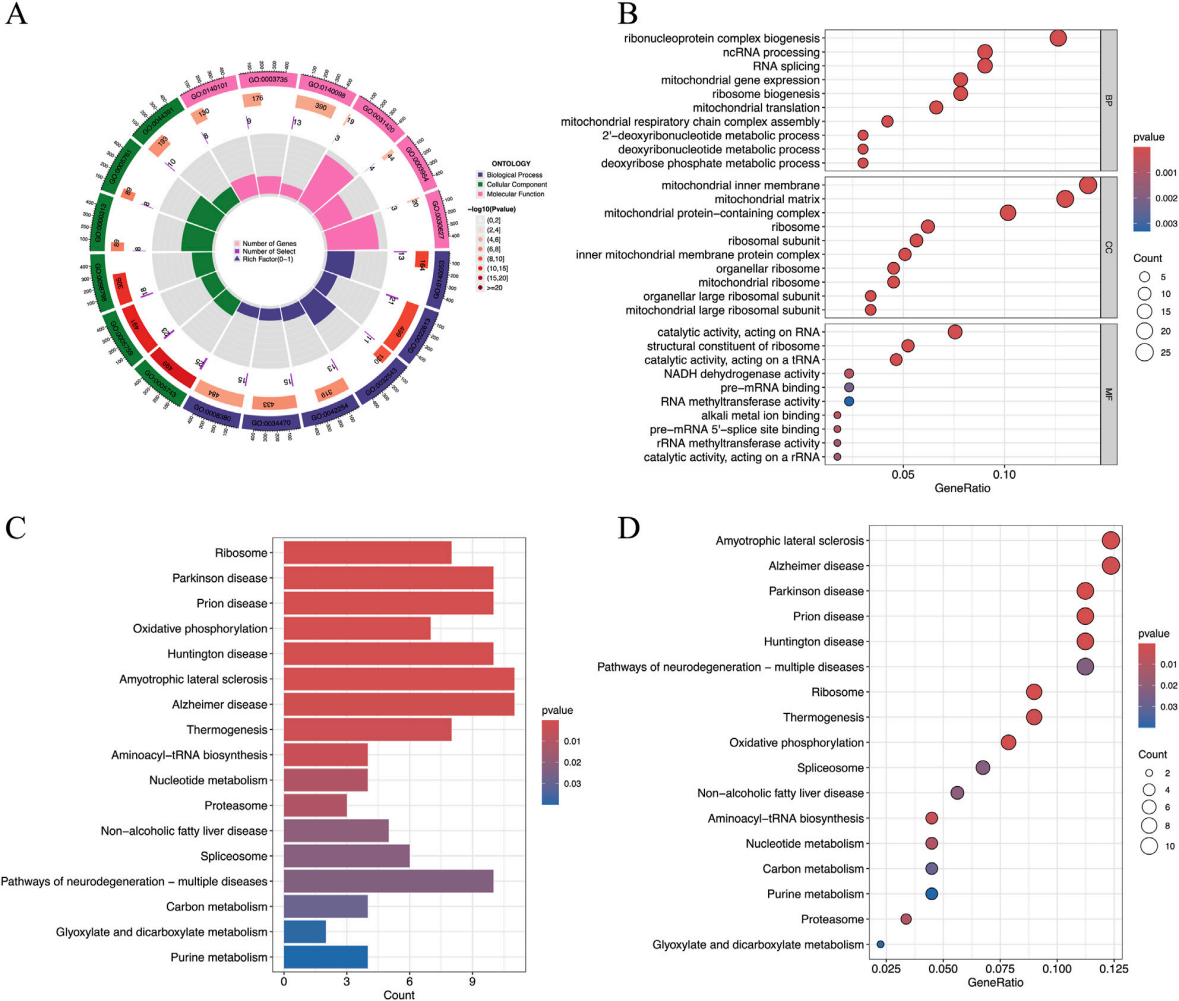
Results of the GO and KEGG analyses of hub genes. **(A)** The results of the GO analysis were shown by a circle diagram, **(B)** The results of the GO analysis were shown by a bubble diagram, **(C)** The results of the KEGG analysis of the hub genes were shown by a bar diagram, and **(D)** The results of the KEGG analysis of the hub genes were shown by a bubble diagram. ; GO, gene ontology; KEGG, Kyoto encyclopedia of genes and genomes.

The GO and KEGG analyses were performed based on intersected genes from WGCNA and DEGs. In this study, the GO analysis focused on three categories, including biological process (BP), cellular component (CC), and molecular function (MF), which represent three different aspects of gene functions. Figure 4A is a brief summary of the results of the GO analysis in terms of a circle diagram. The circle diagram consists of four different circles from the outer to inner circles, which represent GO ID, the number of genes enriched in the GO ID, the number of genes enriched in the GO ID based on the dataset, and the percentage of genes enriched in the dataset. As shown in Figure 4B, the top 10 categories for BP included ribonucleoprotein complex biogenesis, ncRNA processing, RNA splicing, mitochondrial gene expression, ribosome biogenesis, mitochondrial translation, mitochondrial respiratory chain complex assembly, 2′-deoxyribonucleotide metabolic process, deoxyribonucleotide metabolic process, and deoxyribose phosphate metabolic process. Figures 4C,D show the results of the enrichment of signaling pathways participated by hub genes in terms of bar plot and bubble plot, respectively. The top 10 enriched signaling pathways include ribosome, Parkinson’s disease, Prion disease, oxidative phosphorylation, Huntington’s disease, Amyotrophic lateral sclerosis, Alzheimer’s disease, Thermogenesis, aminoacyl-tRNA biosynthesis, and nucleotide metabolism Figure 4C.

### Protein-protein interaction network construction and extraction of the core of the network based on identified hub genes

**FIGURE 5:**
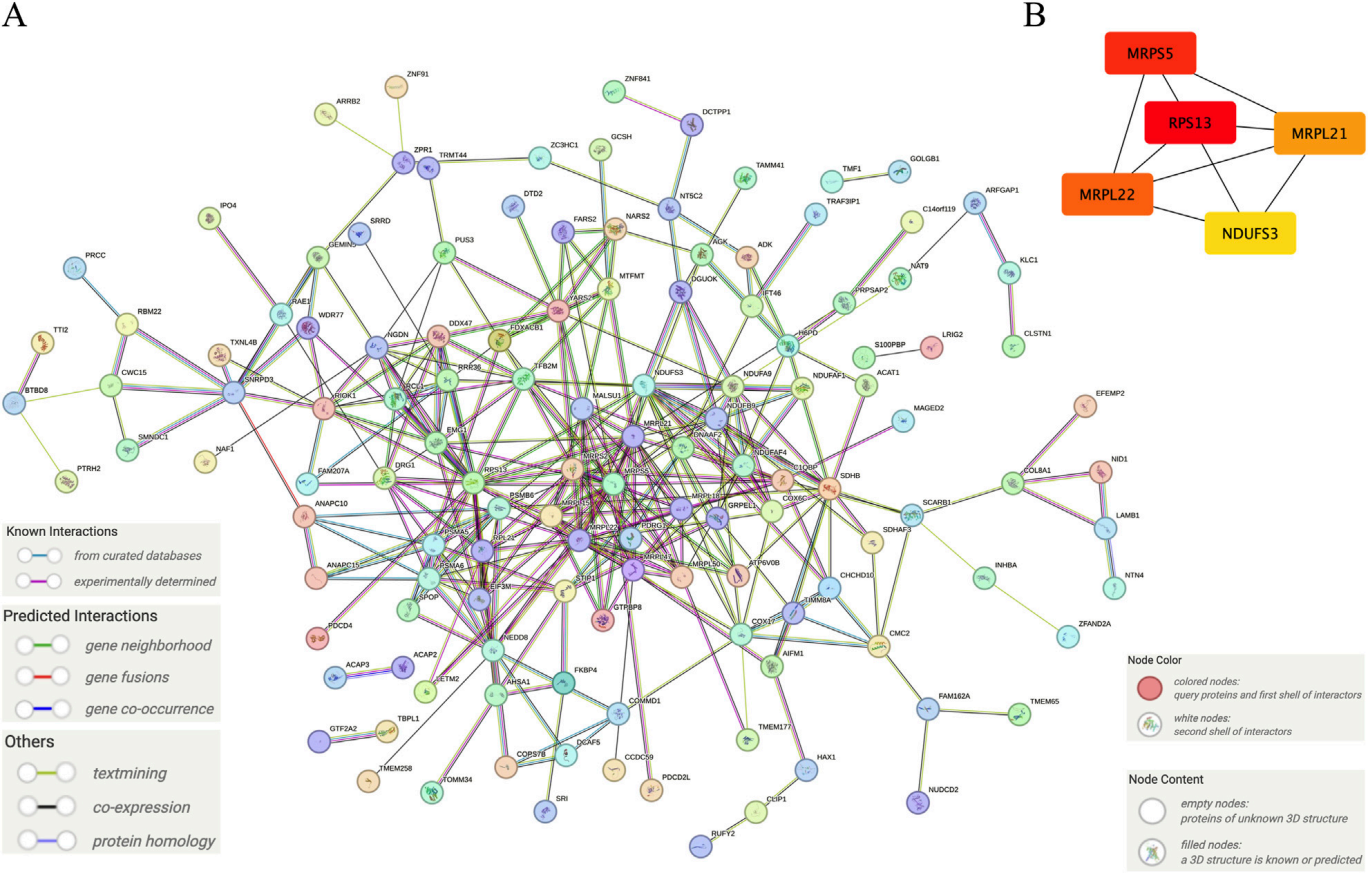
Further screening of the signature genes by protein-protein interaction network. **(A)** The protein-protein interaction network of the hub genes, **(B)** The core network of the protein-protein interaction network of the hub genes. Note: The color in Panel **(B)** was labeled depending on the degree score ranking by Cytoscape. The higher the score, the redder the color. On the contrary, the lower the score, the yellower the color.

Figure 5A shows the interaction between proteins of the identified hub genes. Network nodes represent proteins. Specifically, the color nodes represent query proteins and the first shell of interactors, while the white nodes represent the second shell of interactors. For the content in nodes, the empty nodes represent proteins of unknown 3D structure, while the filled nodes represent a 3D structure that is known or predicted. The edges of the network represent protein-protein associations evidence provided by known interactions (Curated databases and experimentally determined), predicted interactions (Gene neighborhood, gene fusions, and gene co-occurrence), and others (Textmining, co-expression, and protein homology). Figure 5B shows the core abstracted from the protein network consisting of Ribosomal protein S13 (RPS13), Mitochondrial ribosomal protein S5 (MRPS5), mitochondrial ribosomal protein L22 (MRPL22), mitochondrial ribosomal protein L21 (MRPL21), and NADH: ubiquinone oxidoreductase subunit S3 (NDUFS3) based on the score ranking (Table 1) assessment in Cytoscape and were used as the final signature genes that may related to FPVE programming. Among these genes, the redder the color they are, the more interaction they have in the network.

**TABLE 1:** Top five important genes obtained by degree score ranking of the network of hub genes by the Cytoscape software.

Name	Score	Rank
RPS13	24	1
MRPS5	22	2
MRPL22	19	3
MRPL21	18	4
NDUFS3	17	5

### The expression and predictive performance of the signature genes

**FIGURE 6:**
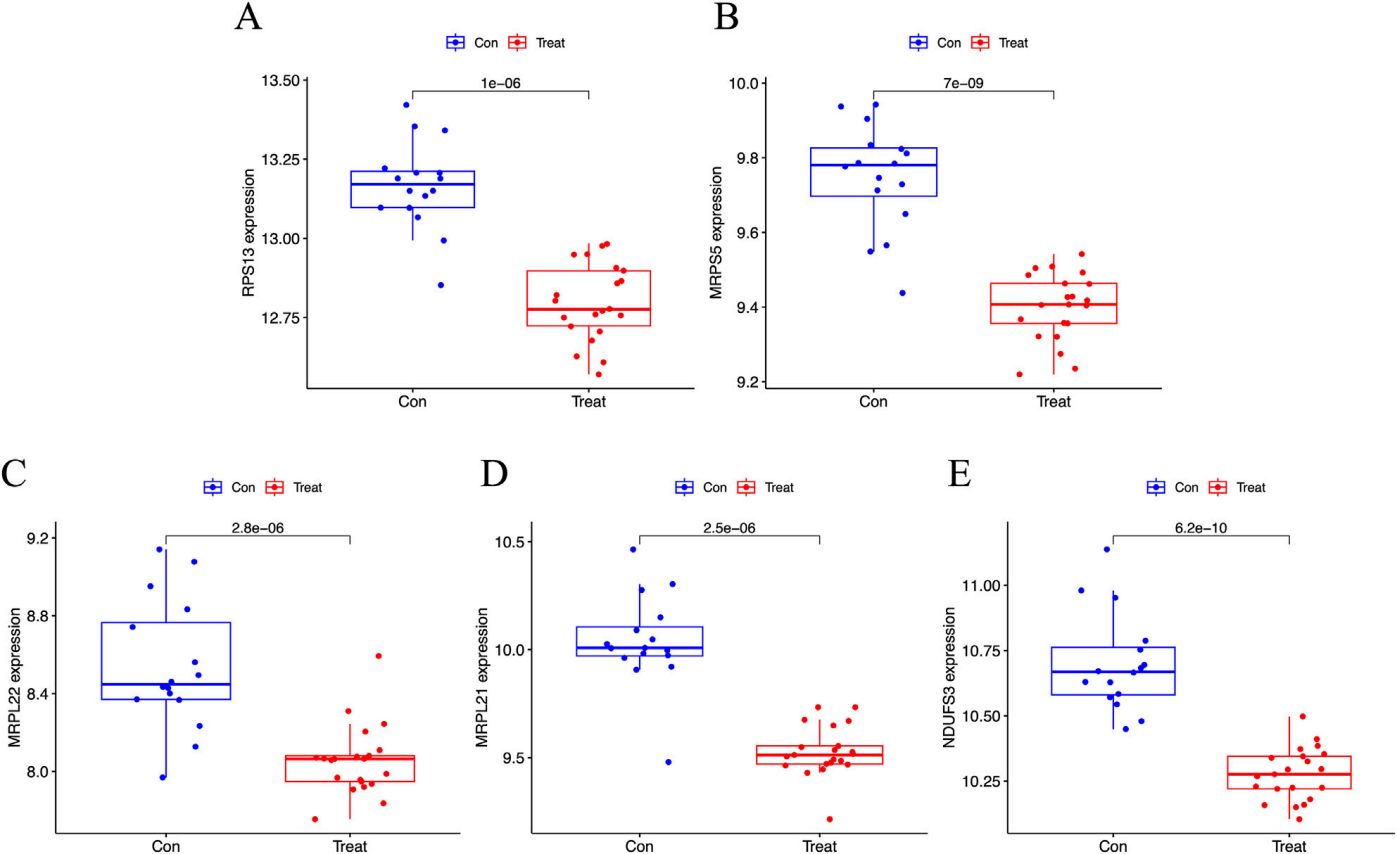
Box plot showing the expression of the signature genes. **(A–E)** Box plot showing the expression of RPS13, MRPS5, MRPL22, MRPL21, and NDUFS3, respectively. RPS13, Ribosomal Protein S13; MRPS5, Mitochondrial ribosomal protein S5; MRPL22, Mitochondrial ribosomal protein L22; MRPL21, Mitochondrial Ribosomal Protein L21; NDUFS3, NADH: Ubiquinone Oxidoreductase Core Subunit S3.

**FIGURE 7:**
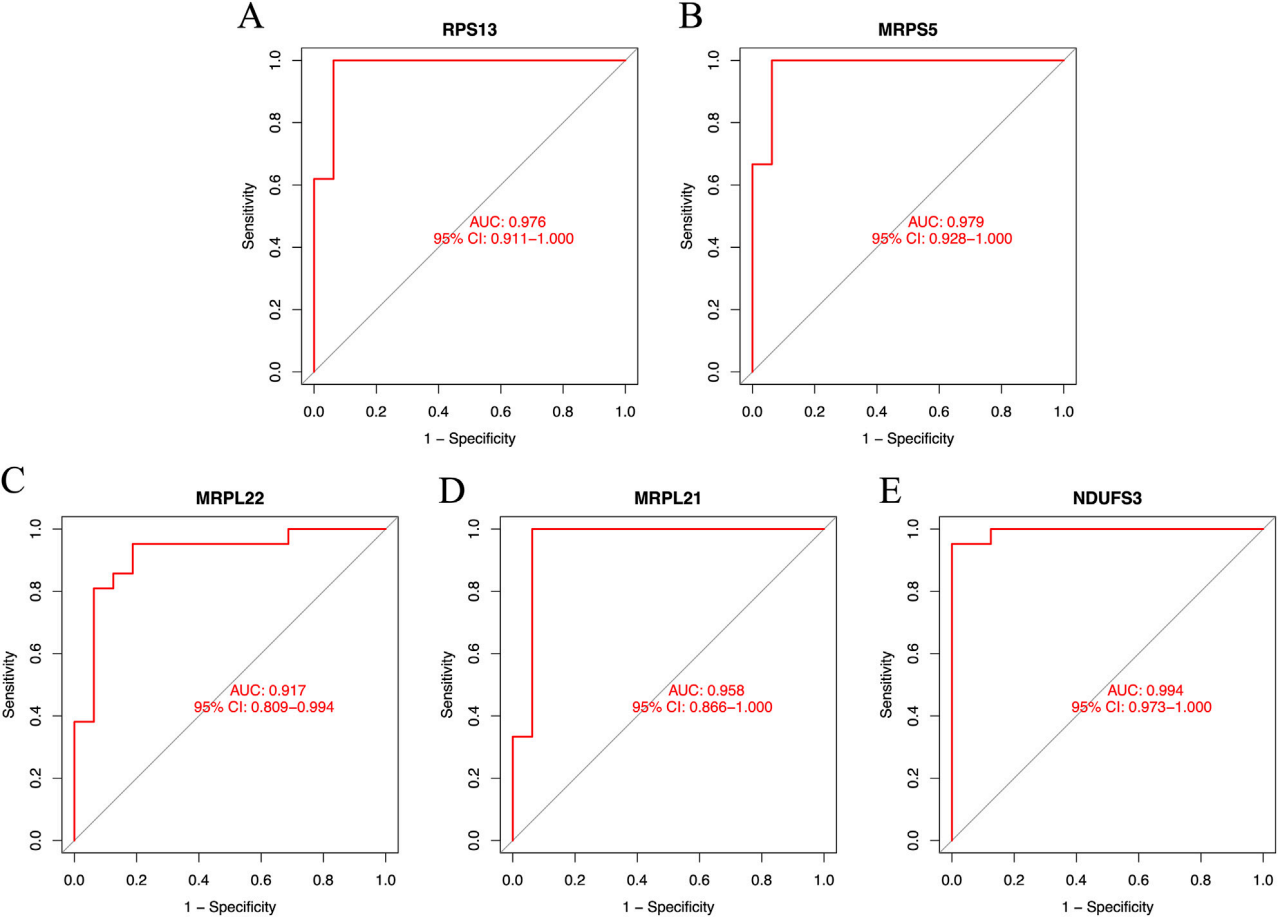
The ROC curves of the signature genes. **(A–E)** The ROC curve for RPS13, MRPS5, MRPL22, MRPL21, and NDUFS3, respectively. ROC, receiver operating characteristic; RPS13, Ribosomal Protein S13; MRPS5, Mitochondrial ribosomal protein S5; MRPL22, Mitochondrial ribosomal protein L22; MRPL21, Mitochondrial Ribosomal Protein L21; NDUFS3, NADH:Ubiquinone Oxidoreductase Core Subunit S3.

The results showed that primary human FPVEs from those women with GDM exhibited significantly lower expression of RPS13, MRPS5, MRPL22, MRPL21, and NDUFS3 compared to those from normal pregnancy (Figures 6A–E). Importantly, we found that these signature genes had an excellent diagnostic performance with the AUC of 0.976, 0.979, 0.917, 0.958, and 0.994, respectively, in the ROC (Figures 7A–E). The significant difference and the high diagnostic performance of the signature genes indicated that they may play crucial roles in FPVE programming and the related gestational complications linked to FPVE dysfunction. The importance of the signature genes makes it necessary to further uncover the signaling pathways and the regulatory network linked to the signature genes.

### KEGG signaling pathway analysis of the signature genes

**FIGURE 8:**
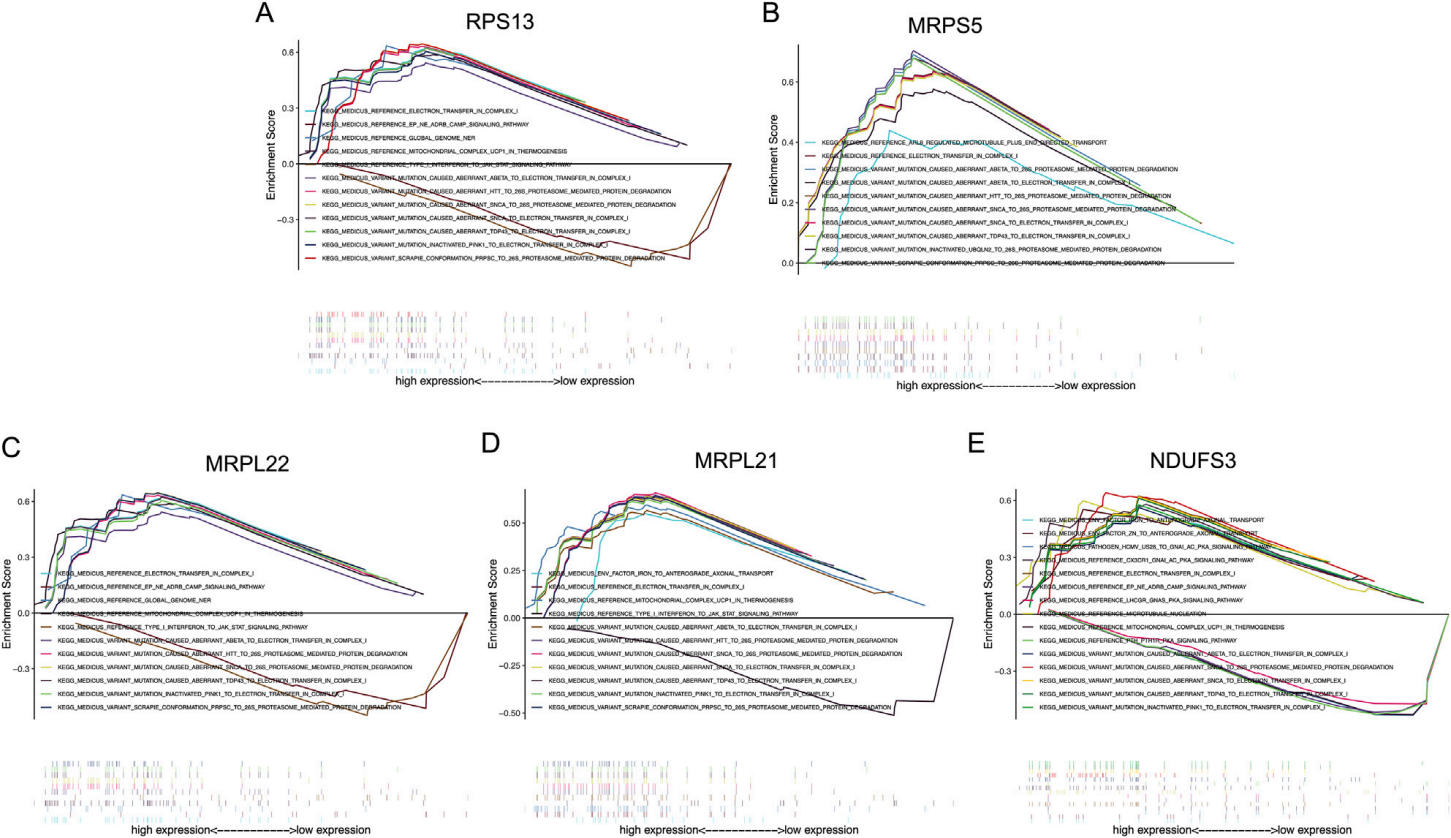
The KEGG analysis of the signature genes. **(A–E)** The possible signaling pathways that RPS13, MRPS5, MRPL22, MRPL21, and NDUFS3 may participate in, respectively. RPS13, Ribosomal Protein S13; MRPS5, Mitochondrial ribosomal protein S5; MRPL22, Mitochondrial ribosomal protein L22; MRPL21, Mitochondrial Ribosomal Protein L21; NDUFS3, NADH:Ubiquinone Oxidoreductase Core Subunit S3.

Signaling pathways involved by these signature genes were studied under the GDM conditions in human vascular FPVEs, and the results are shown in Figure 8. The results showed that RPS13 is involved in electron transfer in complex I, EP-NE-ADRB-CAMP signaling pathway, global genome NER, mitochondrial complex UCP1 in thermogenesis, type I interferon to JAK-STAT signaling pathway, variant mutation caused aberrant a-beta to electron transfer in complex I, variant mutation caused aberrant HTT to 26S proteasome-mediated protein degradation, variant mutation caused aberrant SNCA to 26S proteasome-mediated protein degradation, variant mutation caused aberrant SNCA to electron transfer in complex I, variant mutation caused aberrant TDP43 to electron transfer in complex I, variant mutation inactivated PINK1 to electron transfer in complex I, and variant scrapie conformation PRPSC to 26S proteasome-mediated protein degradation (Figure 8A). MRPS5 participates in ARL8 regulated microtubule plus end-directed transport, electron transfer in complex I, variant mutation caused aberrant a-beta to 26S proteasome-mediated protein degradation, variant mutation caused aberrant a-beta to electron transfer in complex I, variant mutation caused aberrant HTT to 26S proteasome-mediated protein degradation, variant mutation caused aberrant SNCA to 26S proteasome-mediated protein degradation, variant mutation caused aberrant SNCA to electron transfer in complex I, variant mutation caused aberrant TDP43 to electron transfer in complex I, variant mutation inactivated UBQLN2 to 26S proteasome-mediated protein degradation, variant scrapie conformation PRPSC to 26S proteasome-mediated protein degradation (Figure 8B). MRPL22 participates in electron transfer in complex I, EP-NE-ADRB-CAMP signaling pathway, global genome NER, mitochondrial complex UCP1 in thermogenesis, type I interferon to JAK-STAT signaling pathway, variant mutation caused aberrant a-beta to electron transfer in complex I, variant mutation caused aberrant HTT to 26S proteasome-mediated protein degradation, variant mutation caused aberrant SNCA to 26S proteasome-mediated protein degradation, variant mutation caused aberrant TDP43 to electron transfer in complex I, variant mutation inactivated PINK1 to electron transfer in complex I, variant scrapie conformation PRPSC to 26S proteasome-mediated protein degradation (Figure 8C). MRPL21 plays a crucial role in env factor iron to anterograde axonal transport, electron transfer in complex I, mitochondrial complex UCP1 in thermogenesis, type I interferon to JAK-STAT signaling pathway, variant mutation caused aberrant a-beta to electron transfer in complex I, variant mutation caused aberrant HTT to 26S proteasome-mediated protein degradation, variant mutation caused aberrant SNCA to 26S proteasome-mediated protein degradation, variant mutation caused aberrant SNCA to electron transfer in complex I, variant mutation caused aberrant TDP43 to electron transfer in complex I, variant mutation inactivated PINK1 to electron transfer in complex I, variant scrapie conformation PRPSC to 26S proteasome-mediated protein degradation (Figure 8D). NDUFS3 is important in env factor iron to anterograde axonal transport, env factor-Zn to anterograde axonal transport, pathogen HCMV-US28 to GNAI-AC-PKA signaling pathway, CX3CR1-GNAI-AC-PKA signaling pathway, electron transfer in complex I, EP-NE-ADRB-CAMP signaling pathway, LHCGR-GNAS-PKA signaling pathway, microtubule nucleation, mitochondrial complex UCP1 in thermogenesis, PTH-PTH1R-PKA signaling pathway, variant mutation caused aberrant a-beta to electron transfer in complex I, variant mutation caused aberrant SNCA to 26S proteasome-mediated protein degradation, variant mutation caused aberrant SNCA to electron transfer in complex I, variant mutation caused aberrant TDP43 to electron transfer in complex I, variant mutation inactivated PINK1 to electron transfer in complex I (Figure 8E).

### LncRNA-miRNA-target genes interaction network construction

**FIGURE 9:**
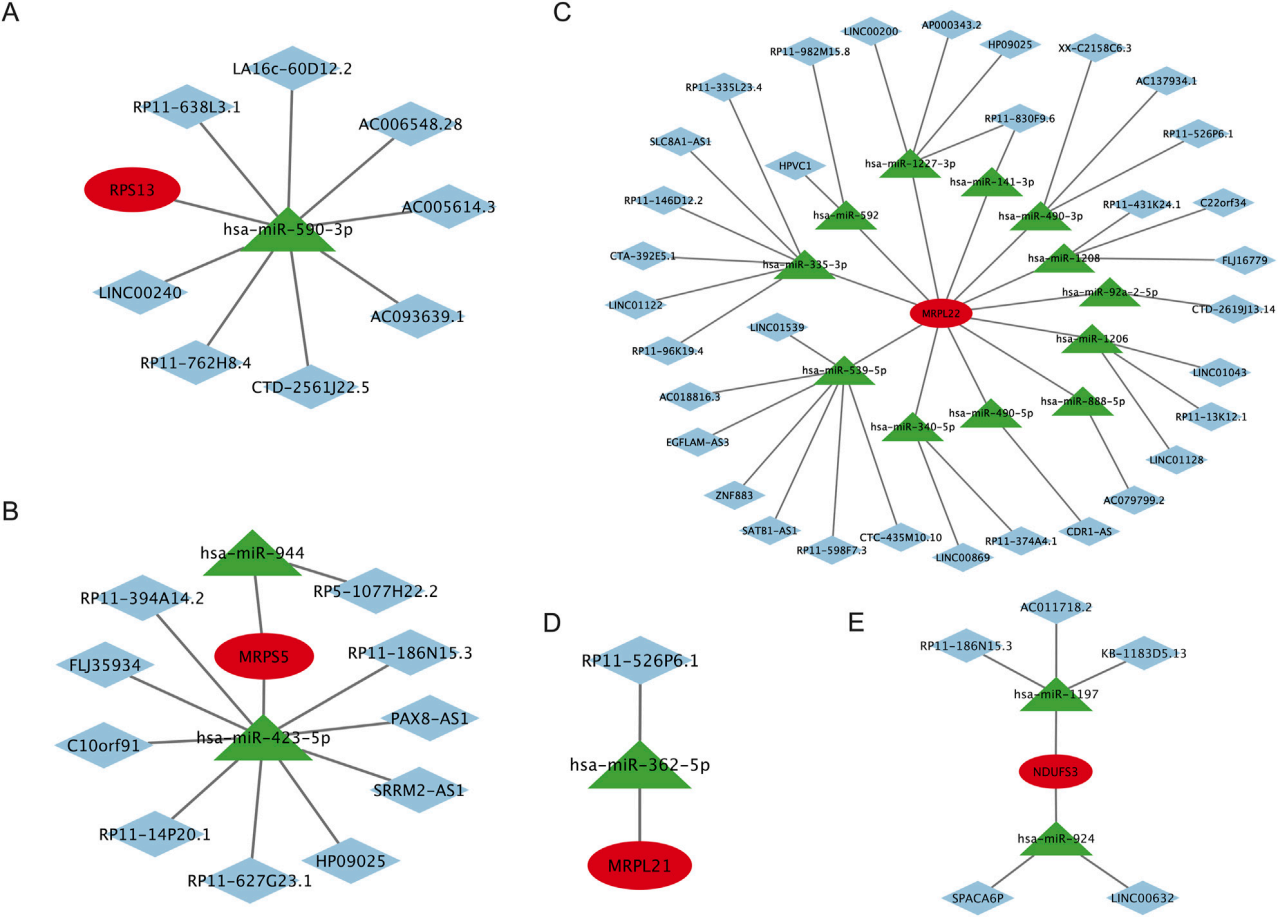
LncRNA-miRNA-target genes interaction network of the signature genes. **(A)** The LncRNA-miRNA-RPS13 interaction network, **(B)** The LncRNA-miRNA-MRPS5 interaction network, **(C)** The LncRNA-miRNA-MRPL22 interaction network, **(D)** The LncRNA-miRNA-MRPL21 interaction network, and **(E)** The LncRNA-miRNA-NDUFS3 interaction network. LncRNA, Long noncoding RNA; miRNA, MicroRNA. RPS13, Ribosomal Protein S13; MRPS5, Mitochondrial ribosomal protein S5; MRPL22, Mitochondrial ribosomal protein L22; MRPL21, Mitochondrial Ribosomal Protein L21; NDUFS3, NADH:Ubiquinone Oxidoreductase Core Subunit S3.

Our results showed that the LncRNA-miRNA-RPS13 interaction network comprises 10 nodes and 9 edges with 8 LncRNAs and 1 miRNA participate in the network (Figure 9A); the LncRNA-miRNA-MRPS5 interaction network comprises 13 nodes and 12 edges with 10 LncRNAs and 2 miRNAs participate in the network (Figure 9B); the LncRNA-miRNA-MRPL22 interaction network comprises 46 nodes and 46 edges with 33 LncRNAs and 12 miRNAs participate in the network (Figure 9C); the LncRNA-miRNA-MRPL21 interaction network comprises 3 nodes and 2 edges with 1 LncRNA and 1 miRNA participate in the network (Figure 9D); and the LncRNA-miRNA-NDUFS3 interaction network comprises 8 nodes and 7 edges with 5 LncRNAs and 2 miRNAs participate in the network (Figure 9E). We can see that the expression of these signature genes is regulated by a number of miRNAs and LncRNAs. For example, the MRPL22 gene is regulated by 12 miRNAs and 33 LncRNAs, and MRPS5 is regulated by 2 miRNAs and 10 LncRNAs. The details of the microRNAs and LncRNAs that are involved in the regulation of signature genes are shown in Figure 9.

## Discussion

This study employed integrated bioinformatics approaches to dissect the molecular mechanisms underlying FPVE dysfunction in GDM. Key discoveries include: Identification of 613 DEGs associated with FPVE programming in GDM, with mitochondrial ribosomal proteins (MRPS5, MRPL21, MRPL22), ribosomal subunit RPS13, and mitochondrial complex I subunit NDUFS3 emerging as pivotal signature genes. Functional enrichment analyses linking these genes to oxidative phosphorylation, ribosome biogenesis, and stress-response pathways, all critical for placental energy metabolism and vascular development. Regulatory network construction reveals complex LncRNA-miRNA-mRNA interactions that post-transcriptionally modulate the expression of these signature genes in GDM.

GDM imposes a hyperglycemic and pro-oxidative milieu on the placenta, disrupting FPVE function, a key interface for maternal-fetal nutrient exchange (Sun et al., 2020; Madazli et al., 2008; Li et al., 2013). Our findings align with this paradigm, highlighting mitochondrial and ribosomal dysfunction as central drivers of FPVE impairment in GDM. The downregulation of mitochondrial ribosomal proteins (MRPS5, MRPL21, MRPL22) underscores their critical role in maintaining placental bioenergetics. These proteins are essential for synthesizing mitochondrial respiratory chain components, and their deficiency in GDM likely impairs electron transport in complex I, reducing ATP production and exacerbating reactive oxygen species accumulation (Zhao et al., 2019). This aligns with prior studies showing that mitochondrial stress in placental endothelial cells correlates with fetal growth restriction (Hu et al., 2020; Burton and Jauniaux, 2018) and gestational hypertension (Vaka et al., 2018; Smith et al., 2021; Opichka et al., 2021; McElwain et al., 2020). For instance, MRPS5 mutations have been linked to mitochondrial translational errors and impaired nucleocytoplasmic communication, mechanisms that may amplify FPVE dysfunction under GDM-induced metabolic stress (Akbergenov et al., 2018).

RPS13, a component of the cytoplasmic ribosome, emerges as a key regulator of FPVE proliferation. Guo et al. (2011) demonstrated that RPS13 suppresses the cell-cycle inhibitor p27 (Kip1) in cancer cells, and our data suggest a conserved mechanism in FPVEs. Reduced RPS13 expression in GDM may elevate p27 (Kip1) levels, arresting endothelial cells in the G1 phase and impairing angiogenesis, a process vital for placental vascular remodeling (Tan et al., 2024). Concurrently, RPS13’s involvement in global protein synthesis highlights its role in maintaining FPVE structural integrity, as disrupted production of junctional proteins could compromise barrier function and nutrient transport.

NDUFS3, a subunit of mitochondrial complex I, bridges energy metabolism and stress resistance. Wang et al. (2024) showed that NDUFS3 activates the AMPK pathway to mitigate oxidative stress, and its downregulation in GDM may blunt this protective response, promoting endothelial apoptosis (Yang et al., 2023; Ma et al., 2023). Intriguingly, our KEGG analysis linked NDUFS3 to neurodegenerative disease pathways (e.g., Parkinson’s disease), raising questions about potential long-term neurological sequelae in GDM-exposed offspring, a hypothesis warranting longitudinal follow-up studies.

We can see that the identified signature genes exhibit striking overlap with molecular pathways implicated in other pregnancy complications. Like GDM, preeclampsia is characterized by placental oxidative stress and mitochondrial dysfunction, suggesting that they may share therapeutic targets for vascular disorders in pregnancy. For fetal growth restriction (FGR), ribosomal dysfunction, as evidenced by RPS13 downregulation, mirrors mechanisms observed in FGR, where impaired placental nutrient transport drives fetal hypoperfusion (Gordijn et al., 2016). This convergence highlights FPVE dysfunction as a common denominator in multiple adverse pregnancy outcomes.

Given the importance of these signature genes, we constructed the LncRNA-miRNA-target gene interaction networks, which may offer valuable perspectives on their regulatory mechanisms. As demonstrated by the networks, these genes are subjected to regulation by multiple non-coding RNAs, creating a complex regulatory framework in which a number of miRNAs and LncRNAs influence each gene. These results emphasize the intricate nature of post-transcriptional regulation in biological systems. Such networks not only deepen our understanding of gene regulation but also highlight potential biomarkers for the early detection of FPVE dysfunction. The insights derived from the interaction network may serve as a useful resource for future investigations into FPVE dysfunction in GDM.

For future research directions, one potential approach to alleviate FPVE dysfunction could be targeting the non-coding RNAs within the regulatory network established by this study to modulate the post-transcriptional expression of these signature genes. Additionally, the development and application of specific medications aimed at enhancing placental bioenergetics, coupled with anti-oxidative damage therapies, may offer promising strategies for mitigating complications associated with GDM.

While this study provides robust bioinformatics insights, several limitations require addressing. On the one hand, this study was performed without external dataset validation because of the lack of appropriate datasets. On the other hand, this study lacks experimental validation. Functional studies in human placental endothelial cell lines, such as HUVECs treated with high glucose, and mouse models of GDM are essential to confirm the causal roles of RPS13 and mitochondrial ribosomal proteins in FPVE dysfunction. Future research integrating these approaches will strengthen the translational impact of our findings, paving the way for precision interventions to improve fetal outcomes in GDM.

## Conclusion

This study establishes mitochondrial and ribosomal dysfunction as central mechanisms driving FPVE programming in GDM. The identified signature genes (RPS13, MRPS5, MRPL21, MRPL22, NDUFS3) not only unravel the molecular cascade linking maternal hyperglycemia to fetal vascular dysfunction but also offer tangible targets for diagnostic and therapeutic innovation. By situating these findings within the broader landscape of pregnancy complications, our work underscores the critical role of FPVE in mediating adverse fetal outcomes and advocates for multi-omics approaches to unravel their complex etiology.

## Data Availability

The original contributions presented in the study are included in the article/Supplementary Material, further inquiries can be directed to the corresponding authors.
